# Workplace interventions that support older employees’ health and work ability - a scoping review

**DOI:** 10.1186/s12913-020-05323-1

**Published:** 2020-05-26

**Authors:** Tina Söderbacka, Linda Nyholm, Lisbeth Fagerström

**Affiliations:** Faculty of Education and Welfare Studies, Åbo Akademi University, Strandgatan 2, 65100 Vasa, Finland

**Keywords:** Health, Intervention, Older employee, Work ability, Scoping review

## Abstract

**Background:**

The aim of this study was to examine workplace interventions that support older employees’ health and work ability and the effect of these interventions.

**Methods:**

We used a scoping review, a type of a systematic literature review in which selected published academic articles and grey literature reports are included, to answer the following questions: 1) What kind of interventions have been made to support older employees’ health? and 2) What effects do these interventions have on older employees’ work ability? The scoping review framework proposed by Arksey and O’Malley and summarized by the Joanna Briggs Institute was used. Four key concepts comprised the basis for the research: health, intervention, older employee and work ability. A total of 8 articles were found to meet the inclusion and exclusion criteria. The study was limited to published academic articles between 2007 and 2019. Participant age varied between 37 and 74 years (overall average age 50–55) and workplaces comprised the intervention settings.

**Results:**

Three main intervention categories were discerned: health checks and counselling for employees on the individual level, interventions based on screenings, and improvements in work environment or organization. Positive behavioral change and lowered health risks can be achieved through health counselling, which increases work ability. Measurements and screenings comprise good ways to chart and follow-up on employees’ work ability and health status. Supervisor training and support from supervisors were seen to have a positive effect on health outcomes and increased work ability.

**Conclusions:**

To guarantee good results, employers should focus on employees’ health and interventions should occur when employees are younger than the studied group. The small number of articles related to intervention studies for the age group studied here indicate that a knowledge gap exists. We maintain that workplaces that promote employees’ health by strengthening older employees’ vitality can encourage employees to have longer careers.

## Background

Between 2010 and 2030, the number of older employees in the European Union (EU) will increase, with the group aged 55–64 years estimated to expand by about 16.2%. Consequently, the mean age of the labor force will rise [1]. Employees’ decreased work ability and/or early work retirement results in high costs for employers and governments alike and leads to inadequate workforce numbers [1]. It is important to support older employees’ health and work ability, both from societal and individual perspectives.

Chronic health problems are related to decreased work ability and lower productivity at work [2]. In Finland in 2018, for example, the average retirement age was 61.3 years and the most common reason for early retirement was muscular-skeletal system problems (33%) and the second most common reason was mental problems (31%). Depression is the main single cause for retirement on a disability pension in Finland [1]. In most EU countries the official retirement age is 65 years, but Spain, Denmark, Germany and France have decided to raise this to 67 years while Great Britain and Ireland have raised this to 68 years [1]. In many countries, for example Finland and Denmark, retirement age is linked to estimated life expectancy [1]. Most EU countries have sought to increase the average/official retirement age and prevent early workforce exits. To guarantee future adequate workforce numbers, employees should already now considerably extend their working careers.

According to Kuoppala, Lamminpää and Husman [3] health promotion is valuable for employees’ well-being and work ability, and they describe health promotion as a process that increases employees’ control of health-related factors and thereby health. Older employees face a number of age-related physical and psychological changes. Between the ages of 40–65 muscle strength declines; by 65 there is a 10–25% decline in muscular capacity compared to the highest capacity [4]. Workplace health promotion should encompass employees’ physical and psychosocial environments [3]. Crawford et al. [4] suggest that occupational health interventions can reduce the risk of early workforce retirement. Job resources, including the possibility to utilize one’s strengths and potential, impact work engagement, which in turn has a positive influence on health [5]. Oakman et al. also suggest that workplace interventions can improve work ability [6].

From the older individual’s perspective, a good working life supports health and work ability [7]. Worsened health has a negative impact on well-being and welfare, because illness results in suffering and decreased income. The maintenance of health, competencies and skills are important motives for prolonging workforce participation and social contacts, financial reward, appreciation and even challenges at work comprise reasons for continuing working [7]. While some research has been conducted, there is a need to summarize the research results available. A scoping review provides an overview through which researchers can disseminate findings and identify knowledge gaps. Therefore, the aim of this study was to examine workplace interventions that support older employees’ health and work ability and the effect of these interventions.

In Finland, the services that occupational health care should provide are delineated by law [8]. The stated purpose of these services is to promote employees’ work ability and functioning, which given the current increased aging of the workforce is a significant task. There is a long tradition behind occupational health care services in Finland, as the first law stipulating these services was confirmed in 1978 [9] and updated in 2001 [10]. In this study, the core elements of occupational health care interventions implemented to support older employees’ health and work ability are defined as: older employee (55 years or older), health, intervention and work ability. When focusing on older employees’ work ability, 55 years or older is a common age criterion; in the EU countries an aging worker is defined as 55–64 years of age [10]. The World Health Organization (1946) defines *health* as a state of complete physical, mental and social well-being and not merely the absence of disease or infirmity. Health is more than a lack of illness; it is understood as a human being’s doing, being and becoming, where the human being’s health resources constitute strong foundations [11]. In this study, *intervention* is defined as an action to improve the work ability of older employees and positively influence, i.e., prolong, workforce participation. *Work ability* here is defined as the balance between work and individual resources [12]. Work ability can be evaluated using the Work Ability Index (WAI), which was developed in Finland in the early 1980s by researchers from the Finnish Institute of Occupational Health [8]. Ilmarinen [8] describes work ability as a house with four floors. The first floor consists of health and functional capacity, which are the basics of work ability. The second floor consists of knowledge and skills, which are a part of work ability. The third floor consists of human resources, comprising values, attitudes and motivation. The fourth floor consists of work, comprising work conditions, contents and demands, community and organization, supervisory work and management. According to Ilmarinen, situated in the immediate surroundings of the “work ability house” are the organizations that support work, such as occupational health care, family and close community, e.g., relatives and friends [8].

## Methods

This study is a scoping review, an increasingly popular method in health sciences used to summarize and synthesize health evidence, and is useful method when charting what is known about a subject. While a scoping review is a type of systematic literature review, its focus is to provide an overview of the research landscape, disseminate findings and identify gaps in the research rather than provide a synthesis or meta-analysis of findings or evaluate research quality [13–15]. A scoping review may therefore draw upon data from any type of research evidence or methodology, and allows researchers to generate findings that can complement clinical trial results [13–15]. Here this method is used to reveal various sources of information, including primary research studies, systematic reviews, meta-analyses and guidelines together with grey literature (materials and research produced by organizations outside of the academic publishing). In the scoping review framework proposed by Arksey and O’Malley [13] and summarized by the Joanna Briggs Institute [14], a six-stage approach to conducting such a review is outlined: (1) identify the research question; (2) identify relevant literature; (3) select the literature; (4) chart the data; (5) collate, summarize and report results; and (6) consultation (stage six is optional and was not used in this review). A scoping review has a broader scope than a systematic review and the purpose of the scoping review is to identify knowledge gaps [16].

### Stage one: identify the research question

Four key concepts comprised the basis for the research: *health, intervention, older employee, work ability*. A two-part research question was developed:

1) What kind of interventions have been implemented to support older employees’ health?

2) Which effects do these interventions have on older employees’ work ability?

### Stage two: identify relevant studies

**Fig. 1:**
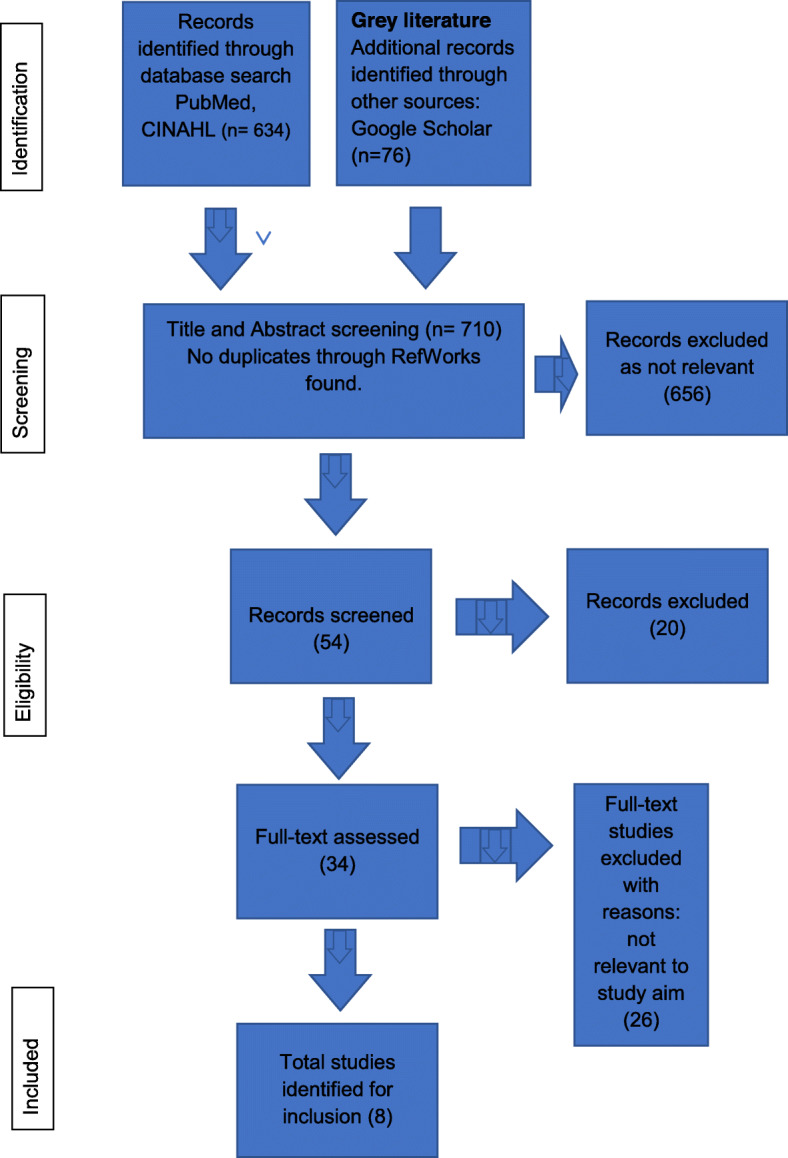
Flow diagram of study selection process

In the identification stage: an initial search for articles published between 2007 and early 2019 was conducted in PubMed and EBSCO (Academic Search Premier, CINAHL, PsycArticles and PsycINFO), using the following search terms and with a focus on older employees: “health”, “intervention” and “older employee”. This initial search resulted in 27 articles from EBSCO and 33,121 from PubMed. In the screening stage: due to the large number of research publications found in PubMed, a second search of PubMed occurred, limited to articles not older than twelve years and with the inclusion of an additional search term, “work ability”. This yielded 607 articles, equating to 634 articles from the first and second database searches. Through other sources (Google Scholar) a further 76 articles were found. No identification of duplicates through RefWorks were found. Of the 710 articles found through the aforementioned searches, title and abstract screening revealed 656 articles that were not relevant, and of the remaining 54 articles a further 20 were excluded. In the eligibility stage these 34 articles were analyzed, resulting in 26 articles being excluded because they were not sufficiently relevant for the study. In the inclusion stage a total of 8 articles were selected for inclusion in accordance with the inclusion criteria. The study selection process is described in a PRISMA flow diagram (see Fig. 1).

### Stage three: select the literature

The 710 articles resulting from the first database, second database and Google Scholar searches were screened for relevance by the first author (TS), who was in turn monitored by the second author (LN). At first only titles and abstracts were reviewed, then full-text articles were assessed, with all study authors thereafter agreeing on which articles were eligible for inclusion. Articles meeting the following were included: presentations of health-supporting interventions predominantly designed for older employees in workplaces. Although this eliminated interventions related to younger employees, it was crucial to achieving a focused search consistent with the study aim. While some of the studies included also encompassed younger participants, all of the studies focused on interventions that prolong workforce participation. The overall average participant age ranged from 50 to 55 years. All three authors together discussed any discrepancies related to inclusion and exclusion. In total, eight studies were included for further in-depth review.

### Stage four: chart the data

The scoping review selection process is shown in Fig. 1. The aim of this study was to examine workplace interventions that support older employees’ health and work ability and the effect of these interventions thus our literature charting emphasized the basic characteristics of each of the articles selected. A data extraction framework was created to present the data in a comprehensive way, including characteristics such as: author, year of publication, origin, aim/purpose, methods, participants, intervention, outcome and key findings (Table 1). The content of the interventions in the included studies was also charted, with regard to similarities and differences. The data in the framework was completed and cross-checked by all authors.

**Table 1:** Comprehensive classification of included articles

Author, Year of publication, Origin	Aim/Purpose	Methods	Participants	Intervention	Outcome and key findings
Ariyoshi (2009) [17], Japan	To evaluate the impact of interventions for menopausal symptoms among employees.	Evaluation included surveys of current employees or former employees with symptoms of menopause and case studies of three women. Interviews with women in their 40s and 50s.	Employees at a newspaper company, employees with symptoms of menopause and case studies of three women.	Development of women’s health management support system, gynecologic check-ups, consultation with the occupational health nurse and occupational physician, etc.	Comparisons before and after implementing changes in the health system revealed that the number of women describing symptoms of menopause decreased from 5 to 0. The number of women retiring or dying while still employed also decreased as well as the number of sick leaves. Interventions for menopause, such as mental health interventions, require specific human resources and systemic support.
Clooster-mans et al. (2015) [18], Nether-lands	To summarize literature on the effects of interventions for ageing workers that address retirement, work ability and work productivity.	A systematic review of four studies.	Workers aged > 40.	Individual (e.g. exercise) programs, workplace programs, personal coach, weekly guided exercise, yoga sessions, free fruit, counselling and education, occupational program for ageing workers by occupational physician, financial support to implement rehabilitation activities.	Limited evidence for a favorable effect on early retirement was found. Workers in the intervention group took later retirement than those in the control group. The risk of early retirement in the plant that received more financial support was about twice as low as the plant with less support.
Costa et al. (2011) [19], Portugal	Goal of creating a decision-making framework oriented toward the maintenance of the health and working ability of aged computer workers.	Assessment of work ability.	Fifty IT workers, mean age 50,8 years old (37–63 years) participated in this study.	Work Ability Index (WAI).	78% of participants had good or excellent work ability and only 2% poor work ability. This study confirms that work ability decreases in workers alongside aging. The evaluation of work ability was elementary to ensuring an age-friendly workplace. The WAI was used to evaluate work ability. The results can help with the early identification of employees with weak work ability, helping to improve working conditions and support the continued working career of workers at their current job.
Kohro et al. (2008) [20], Japan	To screen individuals who are likely to develop lifestyle related diseases and provide early intervention programs.	Review/follow-up study.	Workplace employees and elderly people (65–74 years), all citizens in Japan.	Health campaigns, health checks, counselling intervention. Nationwide program may raise public awareness.	Intervention programs with short follow-up periods are successful regarding cardiovascular risks, but general clinical benefits are observed first about 10 years after intervention.
Koolhaas et al. (2010) [21], Netherlands	To evaluate the process and effectiveness of the intervention compared with care as usual. Research on workplace health promotion.	Cluster- randomized controlled trial design with a 1-year follow-up.	Workers aged 45 years and older; n = workers from intensive care, administration, personnel, executive workers and department supervisors.	Measurements at 3, 6 and 12 months using WAI (work ability), self-reported 12-Item Short Form Health Survey (SF-12; vitality), QQ-method (productivity).	The primary outcomes are work ability, vitality and productivity. The intervention offers a structured method for workers to communicate with their supervisor about their work environment, barriers to work performance and career opportunities.
Strijk et al. (2013) [22], Netherlands	A worksite lifestyle intervention to improve lifestyle behaviors, to keep older workers vital and thereby prolong their labor participation.	A randomized controlled trial design.	367 workers from two academic hospitals. Age ≥ 45 years	6-month intervention: weekly guided group sessions (one yoga, one workout) plus weekly session (aerobic exercise). Individual coach visits aimed at changing workers’ lifestyle behavior. Free fruit provided at guided sessions.	No significant differences in vitality, work engagement, productivity or sick leave were seen between the intervention and control group workers after either 6- or 12-month follow-up. Yoga and workout subgroup analyses showed a 12-month favorable effect on work-related vitality. Implementation of worksite yoga facilities could be a useful strategy to promote vitality-related work outcomes, but only if high compliance can be maximized.
Veller et al. (2007) [23], South Africa	To assess the feasibility and affordability of a targeted screening program for abdominal aortic aneurysms in a group of employer-based medical schemes.	Database review and data extraction, member enrolment by mail. Ultrasound screening.	207 males, 60–65 years. Advice to consult the doctor if they were smoking or had a cardiovascul-ar disease.	Ultrasound screening.	Screening and findings, type and cost of interventions recommended by provider. Screening for abdominal aortic aneurysms reduces morbidity and mortality but at a significant cost; costly intervention.
Wagner et al. (2008) [24], Germany	The effect of cognitive-training programs.	A cognitive-training program was implemented and evaluated.	Middle-aged employees (*n* = 33), 50–59 years, were included in the study at the Psycho-somatic Clinic Bad Neustadt.	Training sessions 7 × 60–90 min, behavioral analysis, behavioral therapy.	Memory performance of the intervention group improved significantly between intake and discharge. A cognitive-training program is useful and effective in patients with mild cognitive impairment.

## Results

Of the eight studies included for in-depth review, five were conducted in Europe, two in Japan and one in South Africa. Participant age varied between 37 and 74 years and workplaces comprised the intervention settings. While in many cases participant profession was not revealed, those included were, among others: office workers, newspaper company management, academic hospital management, intensive care workers, administrative personnel, executive workers and executive worker supervisors. Both women and men were included in all but two studies; one study included only men another only women. Two studies targeted older employees (> 60 years), five studies targeted ageing employees (> 40 years) and one study included employees in a wide age range between 37 and 63 years of age, however the mean age was 50,8 years. A diverse range of research designs were seen: systematic review (1), narrative synthesis (1), statistical analyses of data (2), randomized controlled trial design (2) and others (2).

During the charting of the content of the interventions that support older employees’ health, three main categories based on similarities were identified: “health checks and counselling for employees on the individual level”, “interventions based on screenings”, and “improvements in work environment or organization”. We also present below the effects of interventions on older employees’ work ability in general as seen in the included studies.

### Health checks and counselling for employees on the individual level

In three studies there were interventions with a focus on the individual level, with health checks and counselling interventions. Strijk et al. [22] conducted a six-month intervention study aimed at changing employees’ lifestyles, where employees participated in weekly guided yoga and group workout and aerobic exercise sessions. Ariyoshi [17] conducted an intervention study for women with menopausal symptoms among employees at a newspaper company, where individual consultations with specialists were seen. Kohro et al. [20] investigated an intervention connected to a national health campaign for all Japanese citizens for the prevention of lifestyle-related diseases, including a workplace health check-up program (including individual and group counselling) for employees and a health check-up program for pensioned individuals.

### Interventions based on screenings

In three studies there were interventions based on screenings. The WAI is an instrument used in clinical occupational health and research to assess work ability during health examinations and workplace surveys [10]. Costa et al. [19] used the WAI, with the goal of creating a decision-making health-maintenance framework, to assess employees in the IT sector. Koolhaas et al. [21] used the WAI and self-reported 12-Item Short Form Health Survey (SF-12) to evaluate improvements in health-related outcomes versus care as usual. Veller et al. [23] assessed the feasibility of a targeted screening program for abdominal aortic aneurysms, including ultrasound screening.

### Improvements in work environment or organization

In three studies, there were interventions in which attempts to improve the work environment or organization were investigated. Training supervisors was one intervention used to attempt to prolong workforce participation. Wagner et al. [24] created a cognitive-training program that was implemented on and evaluated by middle-aged employees. Some interventions targeted both the individual and organizational levels. Koolhaas et al. investigated supervisors’ ability to support employees in taking necessary action by enhancing knowledge and competence and better utilization of human resource professionals and occupational health care [21]. In Cloostermans et al.’s [18] systematic review of literature in which the effects of interventions for aging were addressed, a study by Goine et al. [25] was included. An organizational intervention occurred, where workplaces received financial support to implement vocational rehabilitation activities, intended for the improvement of the physical environment [25].

### Effects of interventions on older employees’ work ability in general

Positive behavioral change and lowered health risks can be achieved through health counselling. Kohro et al. [20] found that a nationwide health program may raise public awareness and emphasize that it takes at least 10 years before it is possible to evaluate the benefits of primary prevention. Strijk et al. [22] found that strong participant commitment is necessary, at least regarding yoga as a way to promote vitality at work for aging employees. While in Strijk et al.’s study yoga and workout subgroup analyses showed a 12-month favorable effect on work-related vitality, no significant differences were found in work engagement, productivity or sick leave between the intervention and control group after either 6- or 12-month follow-up [22]. Cloostermans et al. [18] found that the risk for early retirement in the group that received more financial support was about twice as low as the control group with less support. The interventions included individual (e.g., exercise) programs, workplace programs, a personal coach, weekly guided exercise, yoga sessions, free fruit, counselling and education, an occupational program for ageing employees lead by occupational physician, and financial support to implement rehabilitation activities [18]. Differentiating between menopausal disorders and mental health issues is not always easy, and Ariyoshi et al. [17] saw that by implementing changes to the health system, women describing symptoms of menopause, retiring or dying while still employed, and sick leaves decreased.

Wagner et al. [24] found that a cognitive-training program is useful for patients with mild cognitive impairment; the intervention group showed increased results in memory tests and in subjective memory performance and a significantly reduced level of exhaustion. Most of Wagner et al.’s study participants reported that they had better self-confidence in the workplace after training and felt more able to distance themselves from the demands of their jobs [24]. Costa et al. [19] established that the evaluation of work ability is elementary to creating age-friendly workplaces and that the possible identification of employees with weak work ability earlier can improve working conditions and support the continued working career of workers in their current job. They developed interventions based on WAI results (engineering, organizational and training) and found that while the WAI is a useful tool in helping prevent impairments to work for employees on the individual level, there was nonetheless a decrease in the employees’ work ability.

Koolhaas et al. [21] utilized and investigated a structured method whereby employees could communicate with supervisors about their work environment, barriers to work performance and career opportunities. Support from colleagues, informal networking and superiors was seen to be positive, and supervisor training reduced stereotypes, team conflicts and enhanced innovations [21]. Veller et al. [23] found that screening programs, like screening for abdominal aortic aneurysms are costly interventions, but reduce morbidity and mortality [23].

## Discussion

The aim of this study was to examine the workplace interventions that support older employees’ health and work ability and the effect of these interventions. Few studies on such interventions in relation to older employees are seen, and Poscia et al. [26] report that workplace health promotion actions for older employees are generally of poor quality. To guarantee good results, employers should focus on employee health and interventions should occur when employees are younger. One should also remember that employees’ own mental processes regarding retirement, starting about age 55, are important [21]. From a societal perspective, employees’ decreased work ability and/or early work retirement results in high costs for employers and inadequate workforce numbers, while from an individual perspective a good working life supports older employees’ health and work ability. We discerned three main categories based on similarities and found that health promotion interventions are positive for older employees.

Health checks and counselling for employees on the individual level can support older employees’ work ability. Health campaigns that focus on the health and work ability of older employees might be costly, but early retirement due to poor work ability is even more expensive and is often the result of employees suffering from pain or mental illness. The continuous follow-up of employees’ work ability through measurements and screenings provide occupational health care services with information through which to predict risks for early retirement, and we saw that health counselling can lower health risks. Interventions based on measurements and screenings were also seen to support older employees’ work ability. While measurements and screenings comprise good ways to chart and follow-up on employees’ work ability and health status, a plan whereby results are analyzed is nonetheless necessary, because otherwise important data are wasted. Casual screenings appear to be an expensive way of collecting health status data.

Improvements in work environment or organization were even seen to support older employees’ work ability. Possibility to communicate with supervisors was seen to have a positive effect on health outcomes and increased work ability [21]. Other interventions required very long-term study participation, which is often not realistic for participants. It seems that by influencing management and the workplace it is possible to prolong workforce participation. Focus cannot merely be placed on the employee; also, management and organizational changes, including personal consultation for employees seemed to be effective ways to prolong workforce participation and promote better employee working ability.

While ageism and a lack of recognition, development possibilities and predictability are associated with older male employees’ plans for early retirement, work ability is seen to have the strongest association with retirement plans for both genders [27]. Interventions that focuses on management changes and employee support are important, but employees’ work ability is the main resource. Also, Torp and Vinje [28] find that sustainable production is based on workers’ health. Motivation and engagement in work also influences work ability [28].

Many employees consider their functional limitations to be a part of normal aging. Steenstra et al. [29] recommend that multi-component interventions including health service, coordination of services and work modifications could be used to strengthen older employees’ work participation. Honkonen et al. find that work ability meetings could provide a forum to discuss workplace adjustments that support employees in remaining at work, including, e.g., work modifications, adjustments and vocational rehabilitation [30]. Unfortunately, not all employees who would benefit from age-related workplace adjustments find that their needs are being met [31].

We used a scoping review to reveal and describe published main studies related to a topic of interest. We found too few intervention studies in which a focus on older employees was included, and that proper documentation and follow-up was lacking for those interventions that did include older employees. Interventions related to human resources (values, attitudes and motivation) were also not seen. Thus, given such a knowledge gap, we suggest that further, deeper research be undertaken. We found that a scoping review is a useful method when charting knowledge. The strength of the study is that the results can be applied to management and occupational health care in practice. One limitation is that, because in some of the included articles the description of the study method used was ambiguous, it was difficult to categorize the articles. Also, the inclusion and exclusion criteria resulted in a somewhat limited sample size.

## Conclusions

We can conclude based on the results of this scoping review that some positive results are seen from the interventions, though the samples were relatively limited. To guarantee good results employers should focus on employees’ health, and our recommendation is that interventions should occur when employees are younger than the groups studied here. On an individual level, health checks and counselling for employees can support older employees work ability. Screenings provide occupational health care services and management with information through which to predict risks for early retirement. On an organisational level, improvements in work environment were also seen to support older employees work ability. The small number of articles related to intervention studies for older employees indicate that a knowledge gap exists, and further research is needed.

The small number of articles related to intervention studies for older employees indicate that a knowledge gap exists, despite that an ageing population is a challenge for societies throughout the world. We therefore recommend that occupational health and human resources departments implement interventions, such as health campaigns, that promote employees’ work ability as the foundation for their work.

## Data Availability

The datasets used and/or analysed during the current study available from the corresponding author on reasonable request.

## Abbreviations

WAI: Work Ability Index, page 5.
